
JOURNAL INFORMATION
==============================
NLM Title Abbreviation: ScientificWorldJournal
Journal ID: ScientificWorldJournal
NLM Journal ID: 101131163
Journal ID: TSWJ

The Scientific World Journal

ISSN: 2356-6140
EISSN: 1537-744X
Publisher: Wiley

ARTICLE INFORMATION
==============================
PMCID: PMC5936552
PMID: 29861683
DOI: 10.1100/tsw.2005.110
Article ID: 451838
Article version: 1
Subjects: Editorial

Suicide from an international perspective

Merrick Joav 1 2 3 4 *
Zalsman Gil 5
1 National Institute of Child Health and Human Development, Faculty of Health Sciences, Ben Gurion University, Israel
2 Center for Multidisciplinary Research in Aging, Faculty of Health Sciences, Ben Gurion University, Israel
3 Division of Pediatrics, Faculty of Health Sciences, Ben Gurion University, Israel
4 Office of the Medical Director, Division for Mental Retardation, Ministry of Social Affairs, Jerusalem, Israel
5 Child and Adolescent Psychiatry, Geha Mental Health Center and Sackler Faculty of Medicine, Petach Tiqva, Israel
*Joav Merrick: jmerrick@internet-zahav.net
Publication date: 2005
Electronic publication date: 2005 Nov 10
Volume: 5
First page: 888
Last page: 890
Copyright: Copyright © 2005 Joav Merrick and Gil Zalsman.
Copyright year: 2005
License: This is an open access article distributed under the Creative Commons Attribution License, which permits unrestricted use, distribution, and reproduction in any medium, provided the original work is properly cited.
License URL: https://creativecommons.org/licenses/by/3.0/

==============================
Editorial
*TheScientificWorldJOURNAL* (2005) 5, 888–890
ISSN 1537-744X; DOI 10.1100/tsw.2005.110

### Suicide from an International Perspective

Joav Merrick[1,2,3,4,]* and Gil Zalsman[5]

*[1]National Institute of Child Health and Human Development, [2]Center for Multidisciplinary Research in Aging, [3]Division of Pediatrics, Faculty of Health Sciences, Ben Gurion University and [4]Office of the Medical Director, Division for Mental Retardation, Ministry of Social Affairs, Jerusalem, Israel; [5]Child and Adolescent Psychiatry, Geha Mental Health Center and Sackler Faculty of Medicine, Petach Tiqva, Israel*

**E-mail**: jmerrick@internet-zahav.net

*Published November 10, 2005*

### INTRODUCTION

A recent excellent review on adolescent suicide by Keith Hawton and Antony James in the *British Medical Journal*[1] has again focused on the serious public health problem that plagues our society and disrupts families. Suicide is now the third leading cause of death among adolescents and young adults in the age group of 15–24 years[2,3]. The rate of suicide among those from 15–24 years of age in the U.S. has increased from 2.7 per 100,000 in 1950 to 13.2 per 100,000 in 1990[2]. There seem to be many reasons for this fivefold increase, but increased substance abuse, television and video violence, and the easy access to firearms look like substantial reasons for this dramatic increase.

Suicidal behavior, sometimes exaggerated through the media, has resulted in 4% of American high school students attempting suicide within the last 12 months and 8% having made an attempt within their lifetime[2]. The biggest increase in the suicide rate has been seen in the 15- to 19-year-old group, but in the last 10 years, the rate among those 10–14 years old has increased over 100%[2].

### SUICIDE WORLDWIDE

The World Health Organization (WHO) studied suicide around the world since 1950[4] and found an overall increase from 10.1 per 100,000 in 1950 to 16 per 100,000 in 1995 in all ages. In 1950, the study was based on data from only 21 countries, but in 1995, data were supplied from 105 countries. This fact, in itself, can make some of the differences in the figures.

Over the years, the trend has shown a predominance of suicide rates of males over females, which has been relatively constant, but with a slight increase from 3.2:1 in 1950 to 3.6:1 in 1995. One exception is rural China, where the female rates are 1.3 times higher than those for males. The highest suicide rates are found in the Baltic region (e.g., Estonia with 64.3 for males and 14.1 for females), but in absolute figures, one-fourth of all world suicides are committed in China and India. China alone accounts for 20% of all suicides in the world.

*Corresponding author.
©2005 with author.

### SUICIDE IN ISRAEL

Since 1955, the total suicide rates in Israel have remained relatively stable with a peak in 1975 (8.4 per 100,000) and a rate of 5.4 in 1996[4]. In 1955, rates for females were highest, but males have had higher rates since then. The male:female suicide ratio has gradually increased to 3.2:1 in 1996 from 1.3:1 in 1960[4]. In 1995, suicide rates increased progressively with aging. The suicide rate for the age group of 15–24 years was 2.9 per 100,000 in 1955, but had increased to 5.0 in 1995.

Information on suicidal behavior of Israeli adolescents can be found in a national survey conducted in 1994[5], where students from grade 10 and 11 were asked about such behavior in the year prior to the study. The study found that 20.6% of the girls and 13.5% of the boys reported having thought seriously about attempting suicide. A suicide attempt had been planned by 8–10%, 6–7% had attempted suicide, and 3–4% had made a suicide attempt that required treatment by a physician, paramedic, or nurse.

Rates in such surveys are much higher than official Ministry of Health registration, but nevertheless should be taken seriously. Students who reported thinking about, planning, or attempting suicide were more likely to report feelings of unhappiness, moodiness, and loneliness.

### FOCUS ON SUICIDE

In addition to the review in the *British Medical Journal*[1], the *International Journal of Adolescent Medicine and Health* also recently published a special issue on suicide in adolescence[6] and *TheScientificWorldJOURNAL* has now gathered a series of papers into a special issue on international aspects of suicide. This special issue consists of review papers[7,8,9,10,11,12,13] on suicide in New Zealand, suicidal behavior among Arab adolescents; adolescents and adults with intellectual disability, holistic aspects, and eating disorders. Original articles[14,15,16,17,18] include those on suicide among persons with mental illness, guided meditation and suicide, cannabis abuse and suicide in Trinidad, trends in adolescent suicide in Hong Kong, and cultural aspects of suicide.

A book will also shortly be published[19] on the international perspective of adolescent suicide.

### CONCLUSIONS

In conclusion, adult and especially adolescent suicide attempts and actual suicide are a public health concern and we agree that all people who have caused self-harm in a serious way should be assessed in the hospital by a competent child, adolescent, or adult psychiatrist in order to conduct a psychosocial assessment and prevent further harm.

### REFERENCES

1. Hawton, K. and James, A. (2005) Suicide and deliberate self harm in young people. *BMJ* **330,** 891–894.
2. Brent, D.A. (1999) Mood disorders and suicide. In *Ambulatory Pediatrics*. 5$^{th}$ ed. Green, M., Haggerty, R.J., and Weitzman, M., Eds. WB Saunders, Philadelphia. pp. 447–454.
3. American Academy of Pediatrics Committee on Adolescence (2000) Suicide and suicide attempts in adolescents. *Pediatrics* **105(4),** 871–874.
4. WHO (1999) Mental and Behavioral Disorders. Figures and Facts about Suicide. World Health Organization, Geneva. WHO/MNH/MBD/99.1.
5. Harel, Y., Kani, D., and Rahav, G. (1997) Health Behaviors in School-Aged Children: A World Health Organization Cross-National Study. JDC-Brookdale Institute, Jerusalem.
6. Zalsman, G. and Merrick, J. (2005) Special issue on suicide and adolescence. *Int. J. Adolesc. Med. Health* **17(3),** 195–311.
7. Shatahmasebi, S. (2005) Suicide in New Zealand. *TheScientificWorldJOURNAL* **5**, 527–534.
8. Morad, M., Merrick, E., Schwarz, A., and Merrick, J. (2005) A review of suicide behavior among Arab adolescents.

*TheScientificWorldJOURNAL* **5**, 674–679.

9. Merrick, J., Merrick, E., Morad, M., and Kandel, I. (2005) Adolescents with intellectual disability and suicidal behavior. *TheScientificWorldJOURNAL* **5**, 724–728.
10. Merrick, J., Merrick, E., Lunsky, Y., and Kandel, I. (2005) Suicide behavior in persons with intellectual disability. *TheScientificWorldJOURNAL* **5**, 729–735.
11. Ventegodt, S. and Merrick, J. (2005) Suicide from a holistic point of view. *TheScientificWorldJOURNAL* **5,** 759–766.
12. Dancyger, I.F. and Fornari, V.M. (2005) A review of eating disorders and suicide risk in adolescence. *TheScientificWorldJOURNAL* **5**, 803–811.
13. Latzer, Y. and Hochdorf, Z. (2005) A review of suicidal behavior in Anorexia Nervosa. *TheScientificWorldJOURNAL* **5**, 820–827.
14. Shahtahmasebi, S. (2003) Suicides by mentally ill people. *TheScientificWorldJOURNAL* **3,** 684–693.
15. Birnbaum, L. and Birnbaum, A. (2004) In search of inner wisdom: guided mindfulness meditation in the context of suicide. *TheScientificWorldJOURNAL* **4**, 216–227.
16. Maharajh, H.D. and Konings, M. (2005) Cannabis and suicidal behavior among adolescents. A pilot study from Trinidad. *TheScientificWorldJOURNAL* **5**, 576–585.
17. Shek, D.T.L., Lee, B.M., and Chow, J. (2005) Trends in adolescent suicide in Hong Kong for the period 1980 to 2003. *TheScientificWorldJOURNAL* **5**, 702–723.
18. Maharajh, H.D. and Abdool, P.S. (2005) Cultural aspects of suicide. *TheScientificWorldJOURNAL* **5**, 736–746.
19. Merrick, J. and Zalsman, G. (2005) *Suicidal Behavior in Adolescence. An International Perspective.* Freund, London.

**This article should be referenced as follows:**

Merrick, J. and Zalsman, G. (2005) Suicide from an international perspective. *TheScientificWorldJOURNAL* **5**, 888–890. DOI 10.1100/tsw.2005.110.

### BIOSKETCHES

**Joav Merrick, MD, DMSc** is professor of child health and human development, director of the National Institute of Child Health and Human Development, and the medical director of the Division for Mental Retardation, Ministry of Social Affairs, Jerusalem, Israel. E-mail: jmerrick@internet-zahav.net. Website: www.nichd-israel.com

**Gil Zalsman, MD,** Deputy Director of Geha Mental Health Center, Chief of Child and Adolescent Psychiatry at Petach Tiqva in Israel affiliated with the Sackler School of Medicine, Tel Aviv University, Israel. E-mail: zalsman@post.tau.ac.il
